# Shenling Baizhu Powder Modulates Gut Microbiota and Bile Acid Signaling to Alleviate Diet-Induced Diarrhea

**DOI:** 10.3390/ph19091362

**Published:** 2026-08-28

**Authors:** Qin Liu, Huiyi Peng, Min Su, Zhoujin Tan, Maijiao Peng

**Affiliations:** 1 School of Pharmacy, Hunan University of Chinese Medicine, Changsha 410208, China; liuqin998@163.com (Q.L.); anapenghuiyi@163.com (H.P.); 2 Hunan Key Laboratory of Traditional Chinese Medicine Prescription and Syndromes Translational Medicine, Changsha 410208, China; 3 Department of Biochemistry and Molecular Biology, Changsha Medical University, Changsha 410208, China; su.min414@163.com; 4 Hunan Provincial Key Laboratory of the Research and Development of Novel Pharmaceutical Preparations, Changsha 410208, China

**Keywords:** shenling baizhu powder, diarrhea, high-fat diet, gut microbiota, bile acid metabolism, farnesoid X receptor, traditional Chinese medicine

## Abstract

**Background**: Shenling Baizhu Powder (SLBZP) is a traditional Chinese medicine formula used to treat diarrhea. This study investigated its therapeutic effects and potential mechanisms in mice with fatigue- and high-fat diet-induced diarrhea, focusing on gut microbiota, bile acid metabolism, and farnesoid X receptor (FXR)-related signaling. **Methods**: Male Kunming mice were used to establish a model of diarrhea, followed by treatment with SLBZP. Fecal moisture content, serum levels of diamine oxidase and liver enzymes, and intestinal and hepatic histopathological changes were assessed. Small-intestinal microbiota composition, bile acid profiles in colonic contents, and FXR-related signaling in the ileum and liver were further analyzed. **Results**: SLBZP significantly reduced fecal moisture content. It also modulated the small-intestinal microbiota, with a significant increase in *Faecalibaculum*. In colonic contents, SLBZP reduced total bile acid levels, increased the secondary-to-primary bile acid ratio, and altered multiple bile acid species. These changes were accompanied by increased ileal FXR expression and changes in downstream regulators involved in hepatic bile acid synthesis. Correlation analysis further showed associations of selected bacterial taxa and FXR protein expression with colonic bile acid profiles. **Conclusions**: SLBZP alleviates diarrhea induced by fatigue combined with a high-fat diet in mice. Its effects may involve coordinated modulation of the gut microbiota, bile acid homeostasis, and FXR-related signaling, providing experimental support for the pharmacological basis of this traditional formula in diet-related diarrhea.

## 1. Introduction

Diarrhea is a common gastrointestinal disease, primarily characterized by an increase in the number of bowel movements and loose or watery stools, which can lead to dehydration and electrolyte imbalance in severe cases [1,2]. Its occurrence is associated with various factors such as poor diet [3], fatigue [4,5], and environmental stress [6]. Fatigue itself has also been associated with alterations in gut microbial composition, intestinal morphology, energy metabolism, and immune function in mice [7]. As research progresses, gut microbiota dysbiosis and abnormal bile acid metabolism are increasingly recognized as key mechanisms in the development of diarrhea. Bile acids (BAs) are synthesized by the liver from cholesterol; once they enter the intestine, they undergo deconjugation, dehydroxylation, redox reactions, and epimerization mediated by gut microbes, producing various secondary bile acids [8,9]. Consequently, the composition and metabolic activity of the gut microbiota directly shape the diversity, concentration, and ratios of the intestinal bile acid pool, thereby modulating the signaling functions of bile acid receptors such as the farnesoid X receptor (FXR). Gut microbiota dysbiosis can disrupt bile acid deconjugation and the conversion of primary to secondary bile acids, impairing intestinal secretion, motility, sensory function, and barrier integrity, and thus promoting diarrhea [10,11]. Clinical studies indicate that patients with diarrhea-predominant irritable bowel syndrome (IBS-D) have elevated levels of primary bile acids in their feces and abnormal gut microbiota structures [10]. In some IBS-D patients, an enrichment of Clostridia species can further interfere with the feedback regulation and excretion of bile acids, worsening metabolic imbalance [11]. Additionally, Lin et al. observed in diarrheal piglet and mouse models that gut microbiota dysbiosis leads to abnormal metabolism of chenodeoxycholic acid (CDCA), suppressing the FXR- sirtuin 1 (SIRT1)–liver kinase B1 (LKB1) signaling pathway in intestinal epithelial cells, which damages the mucosal barrier and facilitates diarrhea [12]. Recent findings from our team further demonstrate that diarrhea induced by a high-fat diet under fatigue conditions in mice is associated with gut microbiota dysbiosis, bile acid metabolic disturbance, and intestinal barrier damage [13]. Therefore, investigating the alterations in gut microbiota and bile acid metabolism during diarrhea and elucidating the underlying mechanisms from a novel perspective may offer new strategies for the prevention and treatment of diarrhea.

Traditional Chinese medicine compound formulas are characterized by multiple components, targets, and pathways, showing promise in combating diarrhea [14,15]. Shenling Baizhu Powder (SLBZP) is a compound preparation formulated from ten Chinese medicinal herbs, including Renshen (Ginseng Radix et Rhizoma), Baizhu (Atractylodis Macrocephalae Rhizoma), Fuling (Poria), Gancao (Glycyrrhizae Radix et Rhizoma), Shanyao (Dioscoreae Rhizoma), Baibiandou (Lablab Semen Album), Lianzi (Nelumbinis Semen), Yiyiren (Coicis Semen), Sharen (Amomi Fructus), and Jiegeng (Platycodonis Radix).

Modern pharmacological studies have found that the main medicinal ingredients and their active components in SLBZP exert certain therapeutic and protective effects on gastrointestinal diseases. The active component of Renshen, ginsenoside Rk1, can alleviate radiation-induced intestinal injury through the phosphoinositide 3-kinase (PI3K)/protein kinase B (AKT)/mechanistic target of rapamycin (mTOR) signaling pathway [16], and ginsenoside Ro can also alleviate colitis induced by *Salmonella* Typhimurium infection [17]. Baizhu can improve diet-induced diarrhea by remodeling the gut microbiota, repairing the mucosal barrier, and inhibiting inflammatory responses [18]; its active component, atractylenolide II, can promote intestinal epithelial cell proliferation and migration in vitro, participating in intestinal epithelial repair [19]. Gancao can alleviate intestinal inflammation in mice by regulating the gut microbiota and inhibiting inflammatory responses [20]. In addition, Poria polysaccharides can improve gut microbiota dysbiosis and mucosal barrier damage in mice with antibiotic-associated diarrhea [21]. Baibiandou extract can improve symptoms in mice with irritable bowel syndrome-like conditions [22]. Shanyao can improve antibiotic-associated diarrhea by regulating the gut microbiota and increasing short-chain fatty acid levels [23]. Our team’s previous research found that SLBZP can improve diarrhea induced by fatigue combined with a high-fat diet, and this effect may be related to the regulation of gut microbiota and its metabolites (such as short-chain fatty acids and bile acids), lipid metabolism, and intestinal mucosal barrier function [24,25]. However, whether these bile acid alterations are associated with FXR-mediated enterohepatic feedback has not been investigated. The above studies provide modern pharmacological evidence for the treatment of diarrhea by SLBZP through multiple components and multiple targets. Nevertheless, the exact mechanisms underlying SLBZP’s therapeutic effects on diarrhea remain to be fully elucidated.

Therefore, the present study aimed to further investigate the associations among gut microbiota, bile acid metabolism, and FXR-related enterohepatic signaling, with particular attention to ileal FXR–fibroblast growth factor 15 (FGF15) and hepatic FXR-related feedback. This study will provide an experimental basis for elucidating the mechanism underlying SLBZP’s intervention in diarrhea and for its further development and clinical application.

## 2. Results

### 2.1. Effects of SLBZP on General Condition of Mice

**Figure 1:**
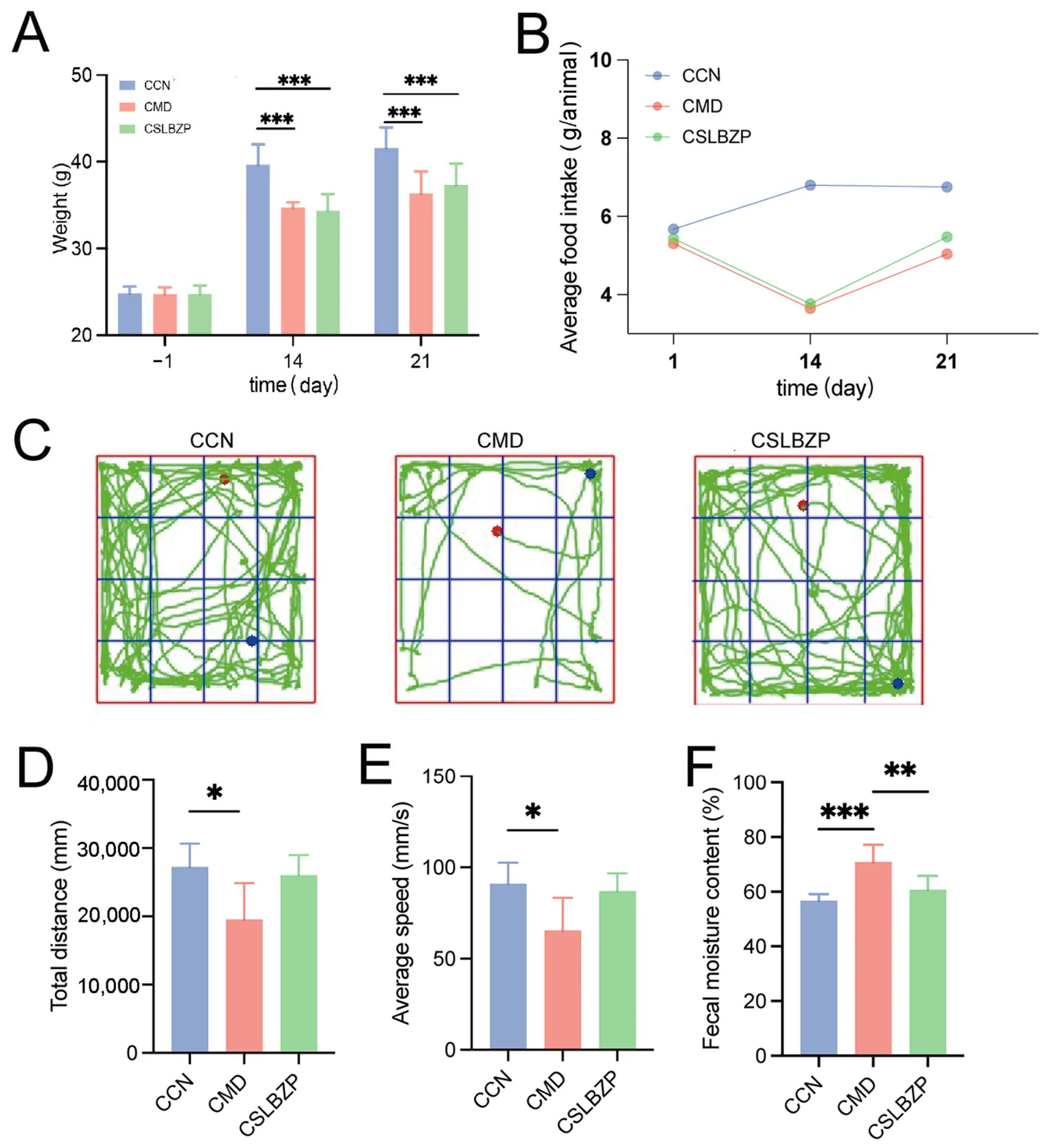
Effects of SLBZP on General Condition of Mice. (**A**) Body weight (*n* = 10 per group). (**B**) Average food intake. (**C**) Representative movement trajectories of mice in the OFT. (**D**) Total distance (*n* = 5 per group). (**E**) Average speed (*n* = 5 per group). (**F**) Fecal moisture content (*n* = 8 per group). Data are expressed as mean ± SD. * *p* < 0.05, ** *p* < 0.01, *** *p* < 0.001.

UHPLC-Q exactive HFX-MS analysis resulted in the putative annotation of 16 constituents in SLBZP. Representative compounds included glycyrrhizic acid, glycyrrhetic acid, ginsenosides Rk1, Ro and F4, atractylenolide II, coumarin, quercetin, and liquiritigenin. The total ion chromatograms acquired in positive- and negative-ion modes are shown in Figure S1, and detailed annotation information is provided in Table S1. After the modeling was completed, the body weights of mice in the model (CMD) group and the CSLBZP group were both lower than those in the normal control (CCN) group (*p* < 0.001), and food intake also decreased. SLBZP treatment did not significantly improve mouse body weight, but food intake showed a slight recovery (Figure 1A,B). The open field test (OFT) results showed that, compared with the CCN group, the CMD group exhibited reduced activity trajectories, total movement distance, and average speed (all *p* < 0.05). After SLBZP intervention, the activity trajectories, total movement distance, and average speed of mice increased to some extent, but the differences were not statistically significant compared with the CMD group (Figure 1C–E). In addition, the fecal water content of mice in the CMD group was higher than that in the CCN group (*p* < 0.001), and it significantly decreased after SLBZP intervention (*p* < 0.01; Figure 1F). These results suggest that SLBZP can alleviate diarrhea in mice.

### 2.2. Effects of SLBZP on Hepatic and Intestinal Histology and Related Serum Biochemical Indices in Mice

**Figure 2:**
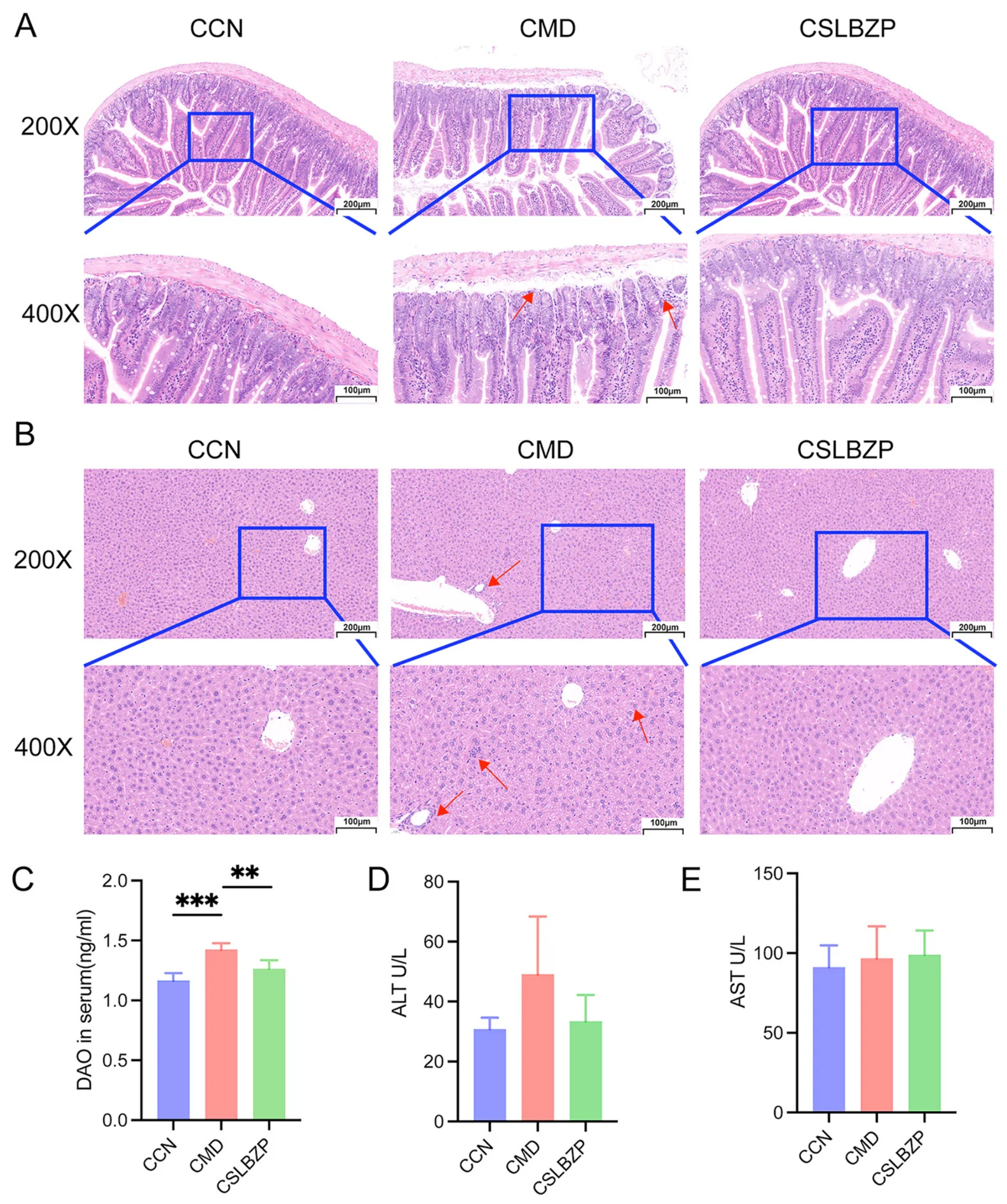
Effects of SLBZP on histopathological alterations and serum biochemical indices in mice. (**A**) Representative H&E staining images of liver tissues at 200× and 400× magnification. (**B**) Representative H&E staining images of small intestinal tissues at 200× and 400× magnification. Red arrows indicate inflammatory cell infiltration. (**C**) Serum DAO concentrations. (**D**) Serum ALT levels. (**E**) Serum AST levels. Data are expressed as mean ± SD (*n* = 5 per group). ** *p* < 0.01, *** *p* < 0.001.

H&E staining results of liver tissue showed that the CCN group had relatively intact liver tissue structure and regularly arranged hepatocytes; in contrast, the CMD group exhibited mild disorganization of liver tissue structure, accompanied by inflammatory cell infiltration (indicated by red arrows). After SLBZP intervention, the above pathological changes were ameliorated (Figure 2A). H&E staining results of small intestinal tissue showed that the CCN group had intact intestinal mucosal structure and relatively regularly arranged villi; the CMD group exhibited disordered villous arrangement with partial damage, reduced goblet cells, and a small amount of inflammatory cell infiltration (indicated by red arrows). After SLBZP intervention, the small intestinal villous structure was improved compared with the CMD group, with more regular arrangement and reduced overall pathological damage (Figure 2B). Consistently, serum diamine oxidase (DAO) levels in the CMD group were significantly higher than those in the CCN group (*p* < 0.001), whereas after SLBZP intervention, DAO levels were significantly reduced compared with the CMD group (*p* < 0.01) (Figure 2C). Serum biochemical results showed that alanine aminotransferase (ALT) levels in the CMD group were numerically higher than those in the CCN group, and showed a decreasing trend after SLBZP intervention, but the differences among groups did not reach statistical significance (Figure 2D). Serum aspartate aminotransferase (AST) levels were generally similar across the three groups, with no statistically significant differences between groups (Figure 2E).

### 2.3. Effects of SLBZP on the Gut Microbiota of Mice

#### 2.3.1. Effects of SLBZP on the Diversity and Composition of the Gut Microbiota

**Figure 3:**
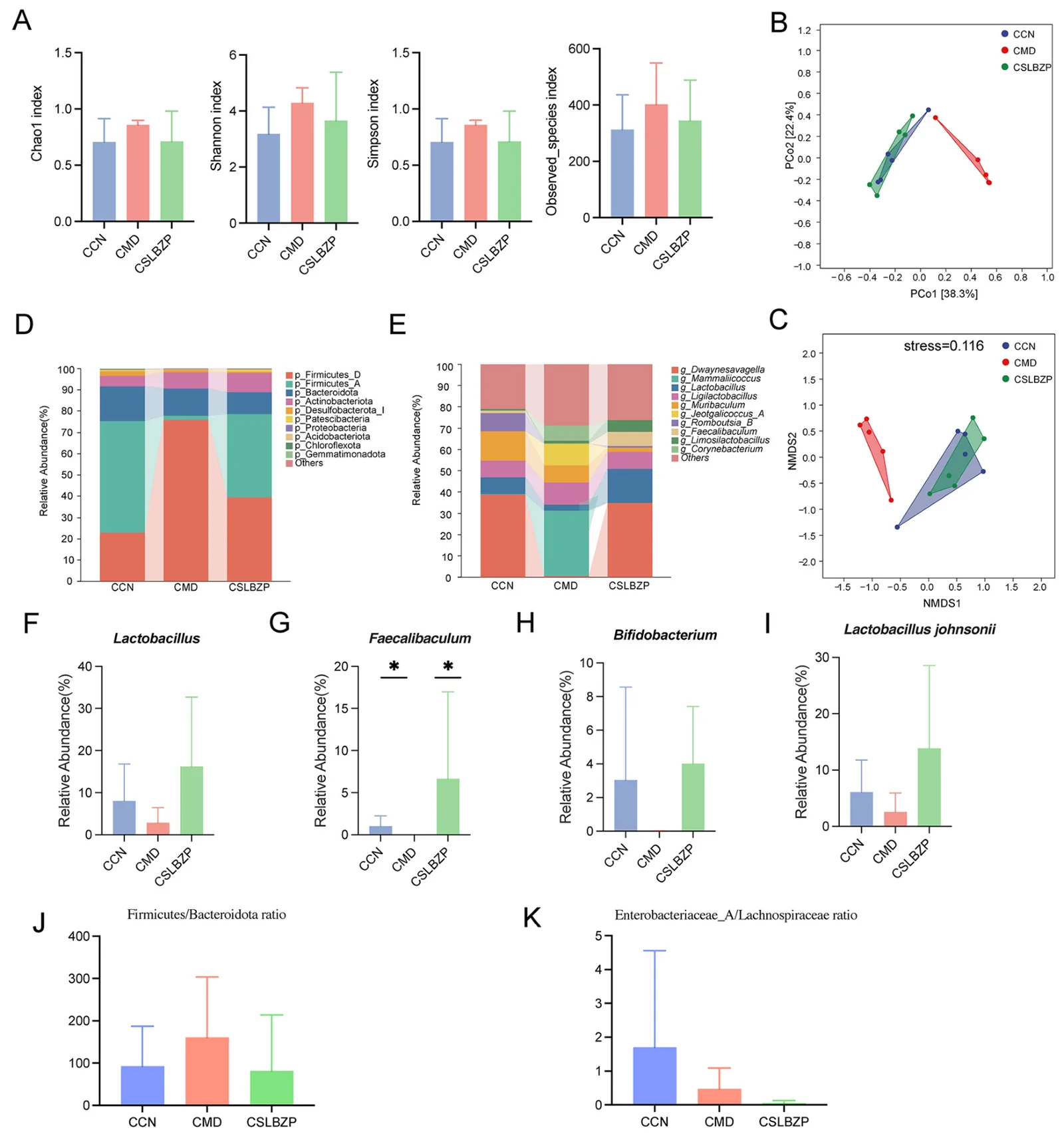
Effects of SLBZP on small intestinal microbial diversity and composition. (**A**) Alpha-diversity indices, including Chao1, Shannon, Simpson, and observed species. (**B**) PCoA. (**C**) NMDS analysis. (**D**) Relative abundances of the dominant bacterial phyla. (**E**) Relative abundances of the dominant bacterial genera. (**F**–**I**) Relative abundances of *Lactobacillus*, *Faecalibaculum*, *Bifidobacterium*, and *Lactobacillus johnsonii*, respectively. (**J**) F/B ratio. (**K**) Enterobacteriaceae_A/Lachnospiraceae ratio. Data in panels A and F–K are expressed as mean ± SD (*n* = 5 per group). * *p* < 0.05.

Alpha diversity analysis indicated that the Chao1, Shannon, Simpson, and Observed species indices of the gut microbiota did not differ significantly among the CCN, CMD, and CSLBZP groups of mice (Figure 3A). Principal coordinates analysis (PCoA) showed that the samples from the CMD group were clearly separated from those of the CCN group, while the samples from the CSLBZP group were distributed closer to the CCN group (Figure 3B). The non-metric multidimensional scaling (NMDS) analysis yielded similar results, with the CMD group showing some separation from both the CCN and CSLBZP groups, whereas the CCN and CSLBZP groups partially overlapped, and the model fit was satisfactory (stress = 0.116, Figure 3C). These findings suggest that fatigue combined with a high-fat diet can alter the overall composition of the gut microbiota in mice, and that SLBZP intervention tended to restore the microbiota structure toward a normal state. The composition of the gut microbiota in each group is shown in Figure 3D,E. At the phylum level, the dominant microbiota mainly included Firmicutes_D, Firmicutes_A, Bacteroidota, Actinobacteriota, Desulfobacterota_I, Patescibacteria, and Proteobacteria (Figure 3D). At the genus level, the dominant taxa mainly included *Dwaynesavagella*, *Mammaliicoccus*, *Lactobacillus*, *Ligilactobacillus*, and *Mucispirillum* (Figure 3E). Compared with the CCN group, the relative abundances of *Lactobacillus*, *Faecalibaculum*, *Bifidobacterium*, and *Lactobacillus johnsonii* were lower in the CMD group but increased after CSLBZP intervention (Figure 3F–I). Among them, the relative abundance of *Faecalibaculum* was significantly lower in the CMD group than in the CCN group and significantly higher in the CSLBZP group than in the CMD group (*p* < 0.05; Figure 3G). No significant differences were observed in the other taxa among the groups. In addition, the Firmicutes/Bacteroidota (F/B) and Enterobacteriaceae_A/Lachnospiraceae ratios were analyzed (Figure 3J,K). The F/B ratio tended to increase in the CMD group and decrease after SLBZP intervention, while the E/L ratio also showed intergroup variation; however, no statistically significant differences were observed.

#### 2.3.2. Effects of SLBZP on Differential Microbiota in Small Intestine Contents

**Figure 4:**
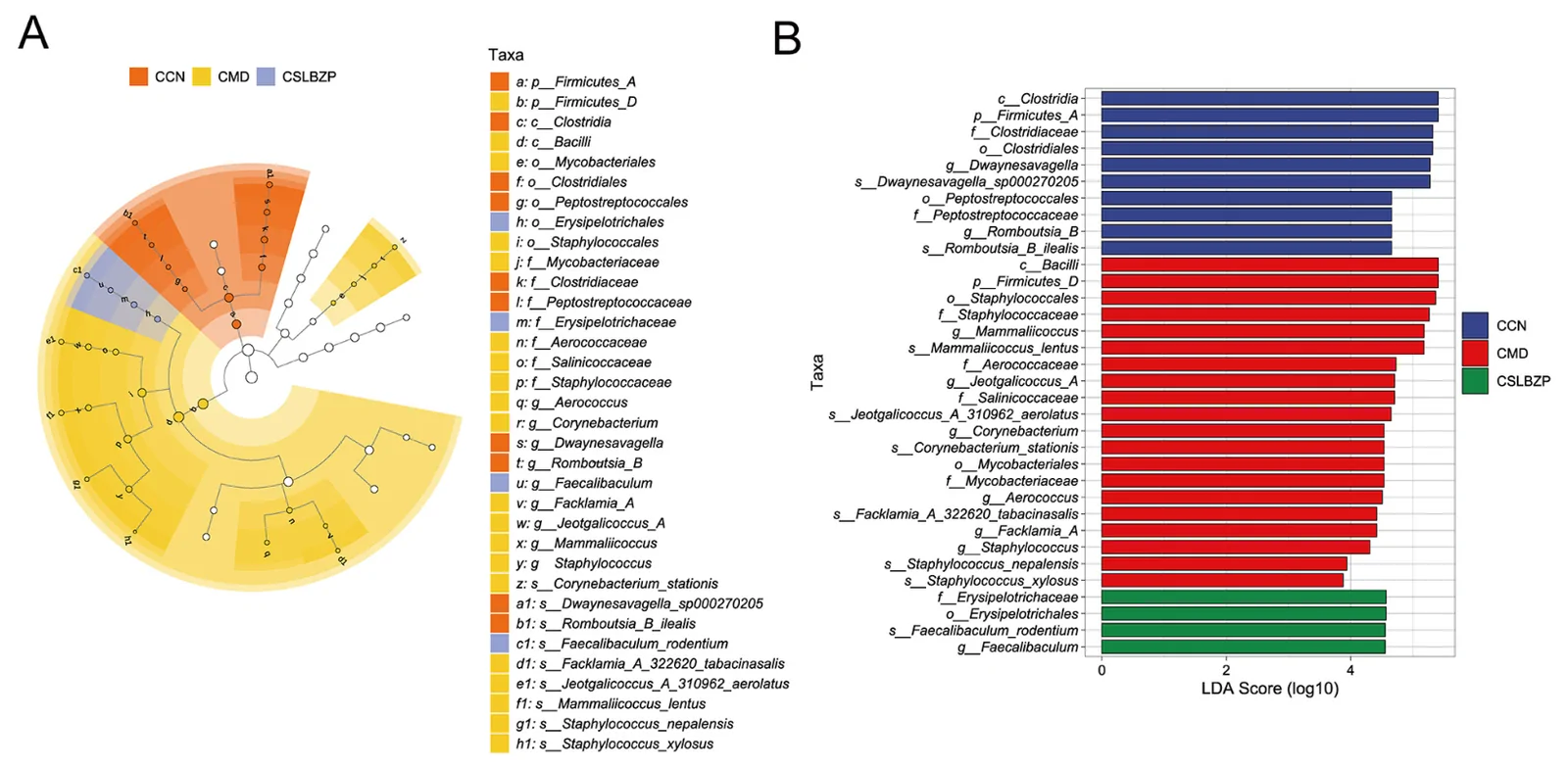
Differentially enriched taxa in the small intestinal microbiota identified by LEfSe analysis. (**A**) Cladogram showing the phylogenetic distribution of taxa enriched in the CCN, CMD, and CSLBZP groups. (**B**) LDA score plot showing the taxa enriched in each group.

Linear discriminant analysis effect size (LEfSe) analysis was used to compare the differential microbiota among the three groups at various taxonomic levels (Figure 4A,B), with the linear discriminant analysis (LDA) score screening threshold set to 2. At the phylum level, the CCN group was mainly enriched in Firmicutes_A, whereas the CMD group was mainly enriched in Firmicutes_D. No significant differential taxa were identified at the phylum level in the CSLBZP group. At the class level, the CCN group was mainly enriched in Clostridia, while the CMD group was mainly enriched in Bacilli and Mycobacteriia. At the order level, the CCN group was mainly enriched in Clostridiales and Peptostreptococcales, the CMD group in Staphylococcales and Mycobacteriales, and the CSLBZP group in Erysipelotrichales. At the family level, the differential taxa in the CCN group mainly included Clostridiaceae and Peptostreptococcaceae; those in the CMD group mainly included Staphylococcaceae, Aerococcaceae, Salinicoccaceae, and Mycobacteriaceae; and the CSLBZP group was mainly enriched in Erysipelotrichaceae. At the genus level, the CCN group was mainly enriched in *Dwaynesavagella* and *Peptacetobacter_B*. The CMD group contained relatively more differential genera, including *Mammaliicoccus*, *Jeotgalicoccus_A*, *Corynebacterium*, *Aerococcus*, *Facklamia_A*, and *Staphylococcus*, whereas the CSLBZP group was mainly enriched in *Faecalibaculum*. At the species level, the differential species in the CCN group mainly included *Dwaynesavagella* sp002702005 and *Peptacetobacter_B muris*. The CMD group mainly included *Mammaliicoccus lentus*, *Corynebacterium stationis*, *Staphylococcus aureus*, and *Staphylococcus xylosus*, as well as species belonging to *Jeotgalicoccus_A* and *Facklamia_A*. The CSLBZP group was mainly enriched in *Faecalibaculum rodentium*.

#### 2.3.3. Effects of SLBZP on Predicted Microbial Functions in Small Intestine Contents

**Figure 5:**
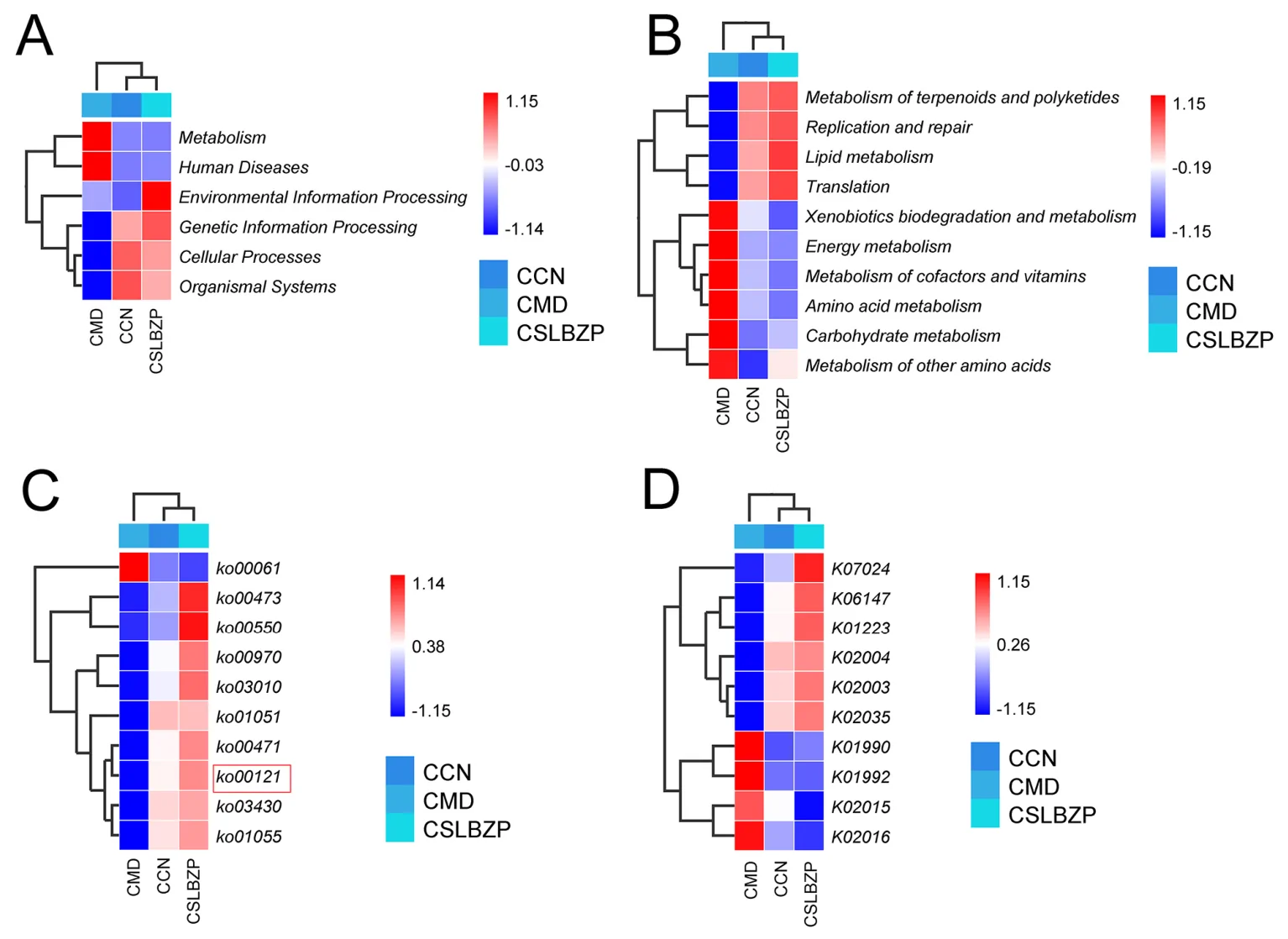
Effects of SLBZP on predicted microbial functions in small intestine contents. (**A**) KEGG level 1 analysis; (**B**) KEGG level 2 analysis; (**C**) KEGG pathway analysis; (**D**) KO analysis.

PICRUSt2-based functional prediction revealed different distributions among the groups at the KEGG level 1, KEGG level 2, pathway, and KO levels (Figure 5). At KEGG level 1, the CMD group showed a distinct predicted functional profile, whereas the profile of the CSLBZP group was more similar to that of the CCN group (Figure 5A). At KEGG level 2, differences were mainly observed in energy metabolism, amino acid metabolism, carbohydrate metabolism, lipid metabolism, replication and repair, and translation (Figure 5B). At the pathway level, differences were observed in fatty acid biosynthesis, D-alanine metabolism, peptidoglycan biosynthesis, aminoacyl-tRNA biosynthesis, ribosome, D-glutamine and D-glutamate metabolism, and mismatch repair (Figure 5C). Notably, the predicted secondary bile acid biosynthesis pathway (ko00121) was less abundant in the CMD group and increased after CSLBZP intervention. Differences in KO terms were also observed among the groups, mainly involving microbial transport and metabolic functions (Figure 5D). These results indicate that CSLBZP intervention was associated with changes in the predicted functional composition of the small intestinal microbiota and partially shifted the functional profile of the CMD group toward that of the CCN group.

### 2.4. Effects of SLBZP on Bile Acid Composition of Mice

**Figure 6:**
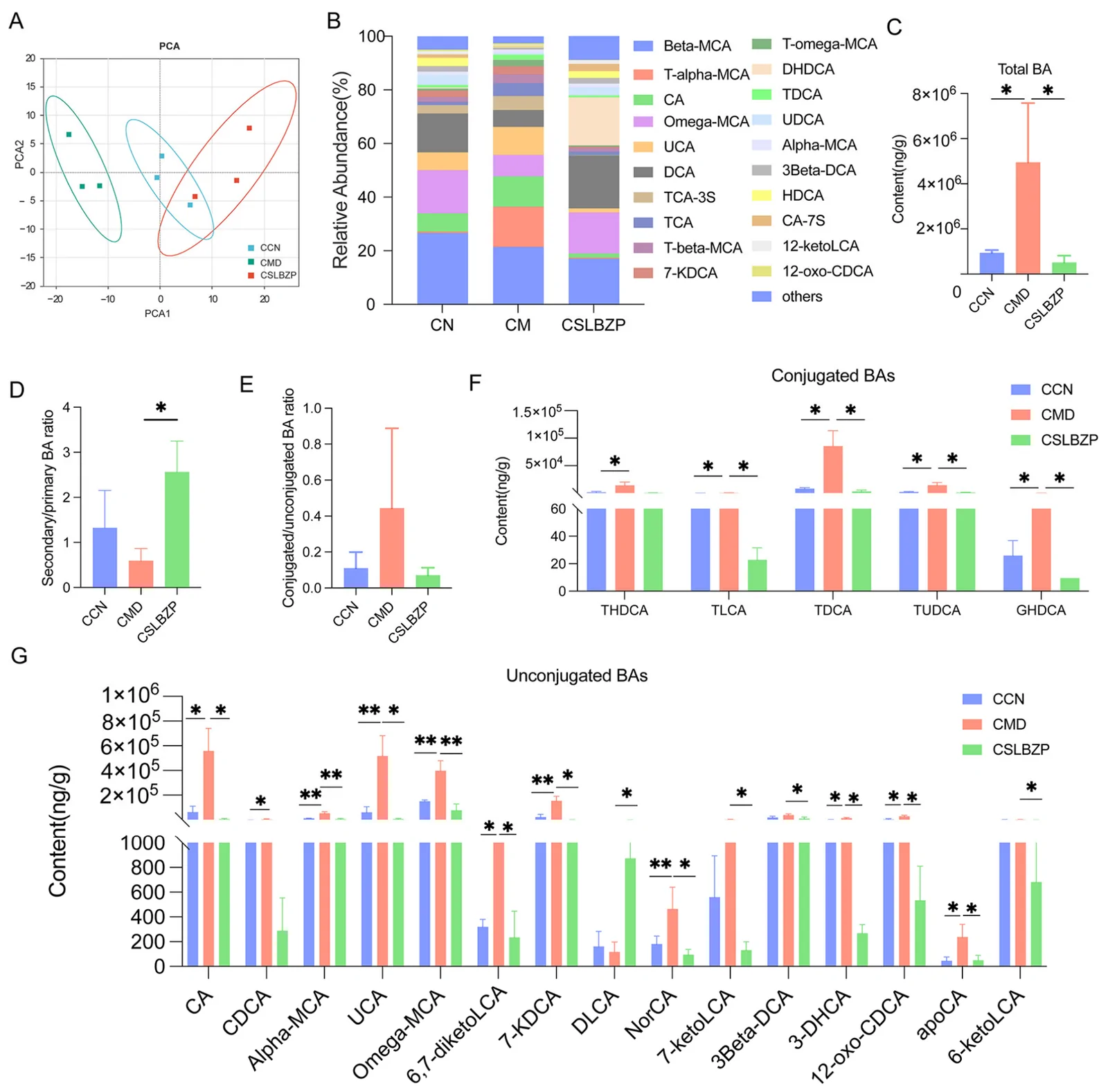
Effects of SLBZP on colonic bile acid profiles in mice. (**A**) PCA score plot. (**B**) Relative abundance of overall bile acid composition in each group. (**C**) Quantification of total bile acid content. (**D**) Secondary/primary bile acid ratio. (**E**) Conjugated/unconjugated bile acid ratio. (**F**) Differential conjugated bile acids among the three groups. (**G**) Differential unconjugated bile acids among the three groups. Data in panels C–G are presented as mean ± SD (*n* = 3 per group). * *p* < 0.05, ** *p* < 0.01.

The PCA results showed an overall separation trend among the three groups of samples, with the CSLBZP group and the CMD group exhibiting relatively distinct separation, while the CCN group and the CSLBZP group displayed partial overlap (Figure 6A). The bile acids in each group mainly included β-MCA, ω-MCA, CA, UCA, DCA, and DHDCA. Compared with the CCN group, the relative abundances of T-α-MCA, CA, and UCA were increased in the CMD group, whereas those of β-MCA, ω-MCA, and DCA were decreased; following SLBZP intervention, the proportions of T-α-MCA, CA, and UCA were reduced, while those of DCA and DHDCA were increased (Figure 6B). Compared with the CCN group, the total bile acid content was significantly increased in the CMD group; after CSLBZP intervention, the total bile acid content was significantly decreased (Figure 6C). The ratio of secondary bile acids to primary bile acids in the CMD group was lower than that in the CCN group, while that in the CSLBZP group was significantly higher than that in the CMD group (Figure 6D). The ratio of conjugated bile acids to unconjugated bile acids showed an increasing trend in the CMD group, but the difference between groups was not statistically significant (Figure 6E). Differential analysis of conjugated bile acids revealed that, compared with the CCN group, the levels of THDCA, TLCA, TDCA, TUDCA, and GHDCA were increased to varying degrees in the CMD group; following SLBZP intervention, the levels of the above bile acids were generally decreased (Figure 6F). Among unconjugated bile acids, the levels of CA, α-MCA, UCA, ω-MCA, 6,7-diketoLCA, 7-KDCA, NorCA, 7-ketoLCA, 3β-DCA, 3-DHCA, 12-oxo-CDCA, and apoCA in the CMD group were increased to varying degrees, and most of them were decreased following SLBZP intervention; DLCA showed an opposite change, being increased in the CSLBZP group (Figure 6G). These results suggest that SLBZP can reduce the total bile acid content in colonic contents, regulate the ratio of secondary to primary bile acids, and modulate the levels of various conjugated and unconjugated bile acids.

### 2.5. Effects of SLBZP on the Expression of Genes and Proteins Involved in Bile Acid Metabolism of Mice

**Figure 7:**
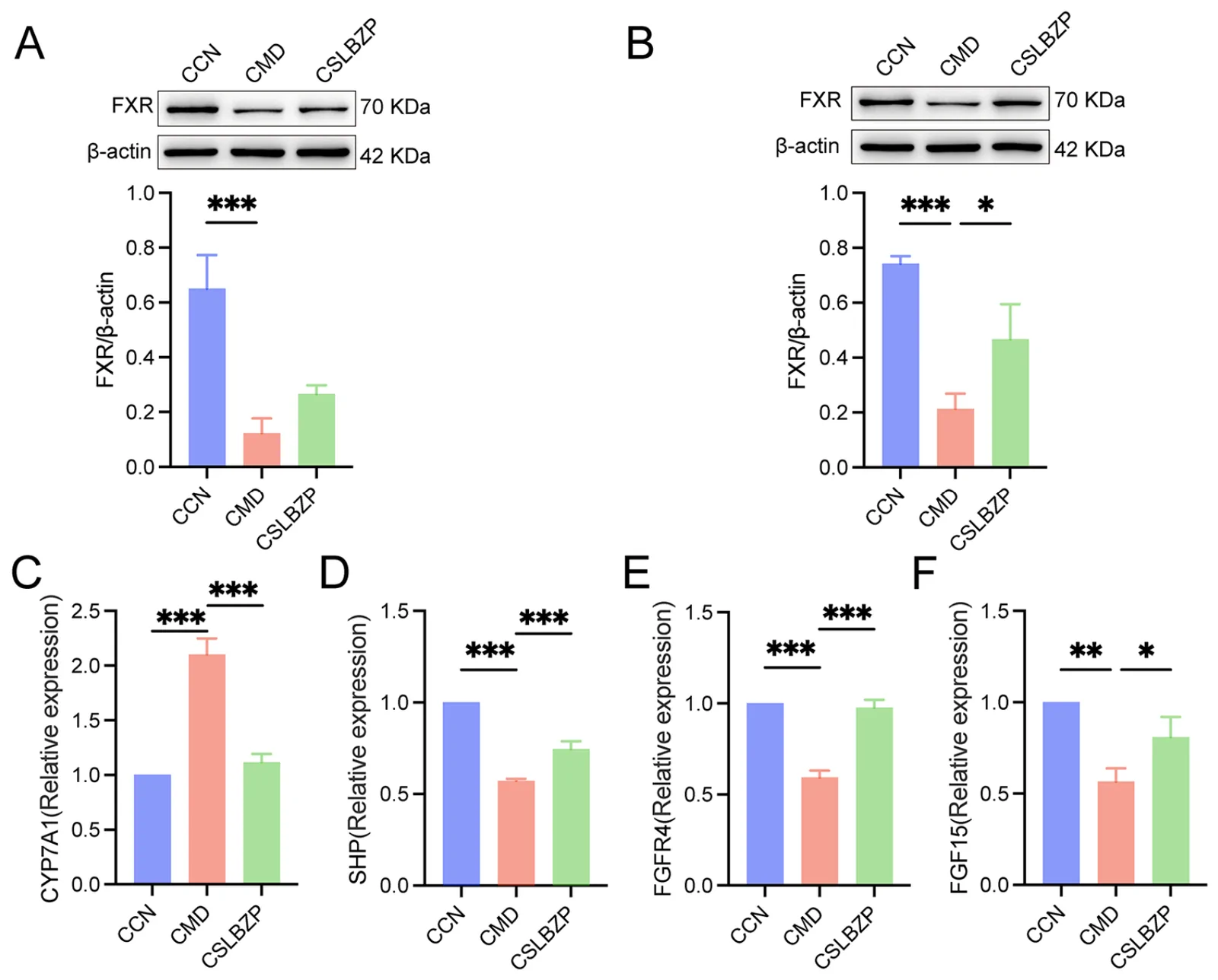
Effects of SLBZP on the expression of genes and proteins involved in bile acid metabolism in mice. (**A**) Representative Western blot bands and quantitative analysis of hepatic FXR protein expression. (**B**) Representative Western blot bands and quantitative analysis of ileal FXR protein expression. (**C**–**F**) Relative mRNA expression of hepatic CYP7A1, hepatic SHP, hepatic FGFR4, and ileal FGF15, respectively. Data are expressed as mean ± SD (*n* = 3 per group). * *p* < 0.05, ** *p* < 0.01, *** *p* < 0.001.

RT-qPCR and Western blot were used to detect the expression of bile acid metabolism-related factors in the liver and ileum. The results showed that, compared with the CCN group, the expression of FXR protein in both the liver and ileum of the CMD group was significantly reduced. After SLBZP intervention, the expression of FXR protein in the ileum was significantly increased, and the expression of FXR protein in the liver also recovered to some extent, but the difference was not statistically significant (Figure 7A,B). In addition, the expression of Cholesterol 7α-hydroxylase (CYP7A1) mRNA in the liver of the CMD group was increased, while the expression of small heterodimer partner (SHP) and Fibroblast growth factor receptor 4(FGFR4) mRNA was decreased, and the expression of FGF15 mRNA in the ileum was downregulated. After SLBZP intervention, the above changes were all improved to varying degrees, manifested as a decrease in CYP7A1 expression and an increase in SHP, FGFR4, and FGF15 expression (Figure 7C–F). This suggests that SLBZP may improve the feedback regulation of bile acid synthesis by modulating the ileal FXR–FGF15 and hepatic FGFR4–SHP–CYP7A1 signaling pathways.

### 2.6. Correlation Analysis

**Figure 8:**
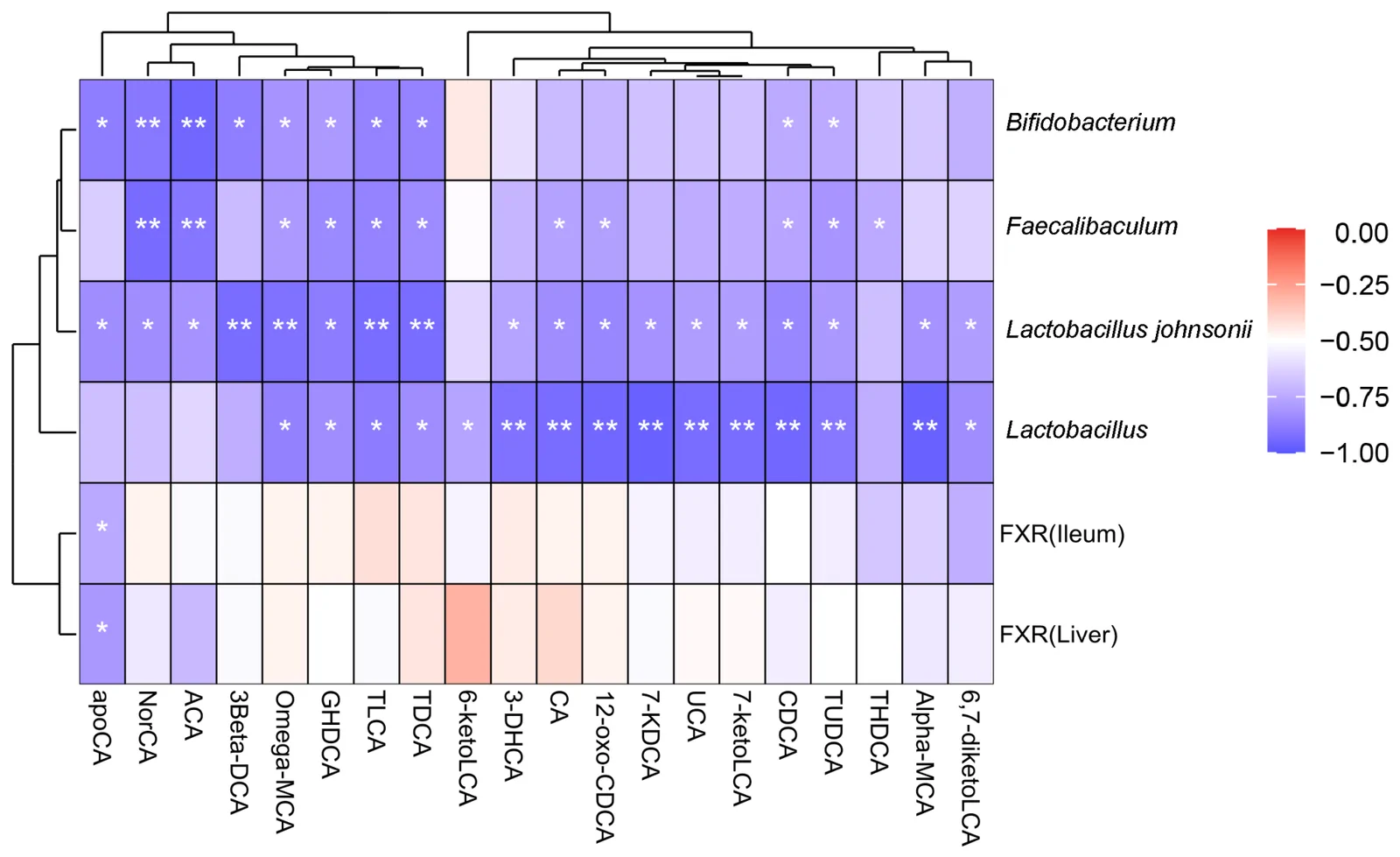
Spearman correlation heatmap of selected small-intestinal bacterial taxa and ileal/hepatic FXR protein expression with colonic bile acid concentrations (*n* = 3 per group). *p* values were adjusted using the Benjamini–Hochberg FDR method. * *p* < 0.05, ** *p* < 0.01.

As shown in Figure 8, correlation analysis was performed among selected small-intestinal bacterial taxa, FXR expression, and colonic bile acid profiles. *Bifidobacterium* was significantly negatively correlated with apoCA, NorCA, ACA, 3β-DCA, ω-MCA, GHDCA, TLCA, TDCA, CDCA, and TUDCA (*p* < 0.05). *Faecalibaculum* showed significant negative correlations with NorCA, ACA, ω-MCA, GHDCA, TLCA, TDCA, CA, 12-oxo-CDCA, CDCA, TUDCA, and THDCA (*p* < 0.05). *Lactobacillus johnsonii* and *Lactobacillus* showed broader significant negative correlations with multiple bile acid species, including ω-MCA, GHDCA, TLCA, TDCA, CA, 12-oxo-CDCA, 7-KDCA, UCA, 7-ketoLCA, CDCA, and TUDCA (*p* < 0.05). In addition, ileal and hepatic FXR showed fewer significant correlations with individual bile acids, and both were significantly negatively correlated with apoCA (*p* < 0.05).

## 3. Discussion

SLBZP is a classic Chinese herbal formula widely used in clinical settings [15], primarily for treating gastrointestinal disorders such as diarrhea [24,25,26,27]. Our previous research has shown that SLBZP can alleviate diarrhea induced by fatigue combined with a high-fat diet, and this effect is linked to changes in gut microbiota and bile acid metabolism [25].

This study employed a mouse model of diarrhea induced by fatigue combined with a high-fat diet to further assess the antidiarrheal efficacy of SLBZP and its potential underlying mechanisms. The results demonstrated that SLBZP intervention significantly reduced fecal water content in the mice, indicating its capacity to alleviate diarrhea in the model. In the OFT, total distance traveled and average speed tended to increase after SLBZP treatment, although these changes did not reach statistical significance. These outcomes are largely consistent with our previous findings [24,25]. DAO is highly active in the intestinal mucosa, primarily in mature intestinal epithelial cells. Serum DAO levels or activity changes are frequently employed as adjunct markers for assessing intestinal mucosal barrier disruption [28,29]. In the present study, model mice exhibited increased serum DAO levels, which were significantly lowered following SLBZP treatment. Together with the H&E staining findings, the significant decrease in serum DAO supports an improvement in intestinal mucosal injury after SLBZP treatment. Liver morphology also showed some improvement, although serum ALT and AST remained unchanged.

Existing studies suggest that the gut microbiota and its metabolites are closely associated with the occurrence of diarrhea [30,31,32]. In this study, after SLBZP intervention, the relative abundances of *Lactobacillus*, *Bifidobacterium*, *Faecalibaculum*, and *Lactobacillus johnsonii* recovered to varying degrees, with the most notable change observed in *Faecalibaculum*. LEfSe analysis also revealed that the SLBZP group was enriched in Erysipelotrichaceae, *Faecalibaculum*, and *Faecalibaculum rodentium* at the family, genus, and species levels, respectively. Previous studies have found that *F. rodentium* participates in the renewal of small intestinal epithelial cells and the maintenance of epithelial homeostasis [33]. In experimental colitis models, enteral nutrition-associated enrichment of *F. rodentium* was also linked to reduced inflammation [34]. Additionally, *Lactobacillus* can contribute to gastrointestinal homeostasis by inhibiting pathogen colonization and producing metabolites such as lactic acid [35]. *Bifidobacterium animalis* subsp. *lactis* XLTG11 can alleviate inflammatory responses in mice with antibiotic-associated diarrhea [36], whereas *L. johnsonii* N5 can ameliorate DSS-induced experimental colitis, maintain intestinal barrier integrity, and reduce gut–liver inflammation [37]. The F/B and E/L ratios can reflect changes in gut microbial community structure. The F/B ratio tended to increase in the model group and decreased after SLBZP treatment, which was generally consistent with changes observed in previous studies of high-fat diet-associated gut microbiota dysbiosis [38]. The E/L ratio also showed a decreasing trend after SLBZP intervention. Previous studies have shown that intestinal inflammation can be accompanied by an increased abundance of Enterobacteriaceae and a decreased abundance of Lachnospiraceae [39]. Therefore, the decrease in the E/L ratio may reflect the modulation of inflammation-associated microbial structure by SLBZP. PICRUSt2 functional prediction further indicated that the predicted abundance of the secondary bile acid biosynthesis pathway (ko00121) was lower in the model group and increased after SLBZP intervention, consistent with the change in the secondary-to-primary bile acid ratio.

In addition to participating in the digestion and absorption of lipids, bile acids also act as signaling molecules to regulate metabolism, immune responses, and intestinal homeostasis [8,40]. After primary bile acids enter the intestine, they are converted into secondary bile acids through deconjugation, oxidation-reduction, epimerization, and 7α-dehydroxylation reactions mediated by the gut microbiota [9]. Under normal conditions, most bile acids are reabsorbed in the ileum and returned to the liver via the enterohepatic circulation [41]. If excessive bile acids enter the colon, they can affect water and electrolyte transport as well as intestinal motility, thereby inducing or exacerbating diarrhea [42,43]. Clinical studies have also found that some patients with diarrhea-predominant IBS-D and chronic functional diarrhea exhibit increased fecal excretion of primary bile acids or total bile acids [10,44,45], indicating that bile acid homeostasis imbalance may contribute to the pathogenesis of diarrhea. The present study found that fatigue combined with a high-fat diet significantly increased the total bile acid content in mouse colonic contents, decreased the ratio of secondary to primary bile acids, and was accompanied by elevated levels of CA, α-MCA, UCA, ω-MCA, and various conjugated bile acids, suggesting colonic bile acid accumulation and abnormal bile acid composition in the model mice. After SLBZP intervention, the total bile acid content in the colon and the levels of various differential bile acids decreased, while the ratio of secondary to primary bile acids increased. These findings indicate that SLBZP may ameliorate bile acid metabolic disturbances in model mice by reducing colonic bile acid accumulation and modulating the composition of the bile acid pool.

Bile acid homeostasis is not solely regulated by the gut microbiota; the feedback loop between the liver and the ileum also plays a crucial role. FXR is a key bile acid receptor in this process [40,46]. In the mouse ileum, stimulation of FXR by bile acids induces the expression of FGF15, which travels through the portal vein to the liver, where it binds to the FGFR4/β-Klotho receptor complex, thereby suppressing CYP7A1-mediated bile acid synthesis [47,48,49,50]. Additionally, hepatic FXR inhibits CYP7A1 transcription by upregulating SHP expression [40,51]. In this study, both hepatic and ileal FXR protein levels were reduced in the model group, along with decreased mRNA expression of ileal FGF15 and hepatic FGFR4 and SHP, whereas CYP7A1 mRNA expression was elevated. These findings suggest that the feedback regulation of bile acid synthesis may be disrupted in the model mice. Following SLBZP intervention, ileal FXR protein and FGF15 mRNA expression increased, as did hepatic FGFR4 and SHP mRNA expression, while CYP7A1 mRNA expression was markedly reduced. These changes are consistent with the observed decrease in total colonic bile acid levels, indicating that SLBZP may restore the feedback regulation of bile acid synthesis. Liu et al. also reported that traditional Chinese medicine compound formulas can enhance bile acid metabolism and reduce intestinal inflammation through modulation of the FXR–FGF15 signaling pathway [52].

Although this study has achieved certain results, there are still some limitations. First, this study only adopted a single dose and did not set up a positive control, so it is not yet possible to determine whether SLBZP has a clear dose–response relationship, nor can it be directly compared with commonly used antidiarrheal drugs. Second, the analysis of the chemical components of SLBZP in this study was mainly based on high-resolution mass spectrometry and database matching, lacking verification by reference standards, and further investigation of components entering the blood or reaching gastrointestinal tissues was not conducted; therefore, the actual material basis for its effects remains unclear. In addition, 16S rRNA sequencing and PICRUSt2 analysis primarily reflect the composition and potential functions of the microbiota and cannot fully replace metagenomic and functional experiments. Due to the small sample sizes for bile acid detection and molecular biology experiments, the current results mainly indicate correlations among changes in the microbiota, bile acid metabolism, and FXR-related signaling but are insufficient to prove direct causal relationships. Future studies could further validate the proposed mechanism by incorporating multiple dose groups and positive controls, conducting targeted verification of key components and bile acids, and employing fecal microbiota transplantation or FXR pathway intervention experiments.

## 4. Materials and Methods

### 4.1. Preparation of SLBZP

The ten herbal materials were combined at a weight ratio of 15:15:15:15:9:12:9:6:6:10. The formulation was based on the classical SLBZP prescription originally recorded in *Taiping Huimin Hejiju Fang*, and its composition and proportions were consistent with those used in our previous studies [24,25]. The composition and dosage of each herbal ingredient in SLBZP are listed in Table 1. All herbal materials were purchased from the First Affiliated Hospital of Hunan University of Chinese Medicine and authenticated by Professor Bingmei Xiao from the Department of Chinese Medicinal Resources, School of Pharmacy, Hunan University of Chinese Medicine, Changsha, China.

### 4.2. LC-MS/MS Analysis of SLBZP

An aliquot of the SLBZP decoction was mixed with methanol–acetonitrile (1:1, *v*/*v*) at a sample-to-solvent ratio of 1:2, vortexed for 60 s, and ultrasonicated for 30 min. After centrifugation at 12,000 rpm for 10 min at 4 °C, the supernatant was incubated at −20 °C for 1 h, centrifuged again, vacuum-dried, and reconstituted in 100 μL of 50% acetonitrile for analysis. Chromatographic separation was performed using a Vanquish ultra-high-performance liquid chromatography system (Thermo Fisher Scientific, Germering, Germany) coupled to a Q Exactive HFX Orbitrap mass spectrometer (Thermo Fisher Scientific, Bremen, Germany) with an ACQUITY UPLC HSS T3 column (100 × 2.1 mm, 1.8 μm, Waters Corporation, Milford, MA, USA). Mobile phases A and B consisted of 0.1% formic acid in water and 0.1% formic acid in acetonitrile, respectively. The flow rate was 0.3 mL/min, the column temperature was 40 °C, and the injection volume was 2 μL. The gradient was programmed as follows: 5% B at 0–1.5 min, 5–30% B at 1.5–15 min, 30–60% B at 15–22 min, 60–100% B at 22–26 min, 100% B at 26–31.5 min, 100–5% B at 31.5–31.6 min, and 5% B at 31.6–35 min. Mass spectra were acquired in positive- and negative-ion modes using Full MS/ddMS^2^ over an m/z range of 70–1050, with resolutions of 70,000 for MS^1^ and 17,500 for MS^2^. Compounds were putatively annotated by matching accurate mass, adduct forms, and MS/MS fragmentation patterns against an in-house traditional Chinese medicine database. Metabolite features with an absolute mass error of ≤10 ppm and an MS/MS spectral matching score of ≥70 were retained.

### 4.3. Animal Experiment

Thirty 4-week-old male Kunming (KM) mice with an initial body weight of 20 ± 2 g were purchased from Hunan SJA Laboratory Animal Co., Ltd. (Changsha, China) Male KM mice were selected because the fatigue- and HFD-induced diarrhea model used in the present study had previously been established and characterized in the same mouse stock by our research group [4,5,13,24,25]. The continued use of KM mice ensured methodological consistency and facilitated comparison with our previous findings. The animals were housed in a specific pathogen-free (SPF) facility at Hunan University of Chinese Medicine under controlled conditions of 23–25 °C, 50–70% relative humidity, and a 12 h light/dark cycle. All mice had ad libitum access to standard chow and drinking water. All animal procedures were approved by the Animal Ethics Committee of Hunan University of Chinese Medicine (approval No. HNUCM21-2025-10; approved on 8 May 2025) and were conducted in accordance with the institutional guidelines for the care and use of laboratory animals.

After a 3-day acclimatization period, the mice were randomly assigned, using a random-number table, to a normal control group (CCN, *n* = 10) or a model-induction cohort (*n* = 20). Mice in the model-induction cohort were subjected to the multiple-platform standing procedure for 4 h/day for 14 consecutive days to induce fatigue. During days 8–14 of model induction, these mice additionally received lard by oral gavage at a total daily dose of 20 mL/kg/day, administered in two divided doses, according to previously established procedures [13]. Mice in the CCN group received an equivalent volume of sterile water by oral gavage during the same period.

**Figure 9:**
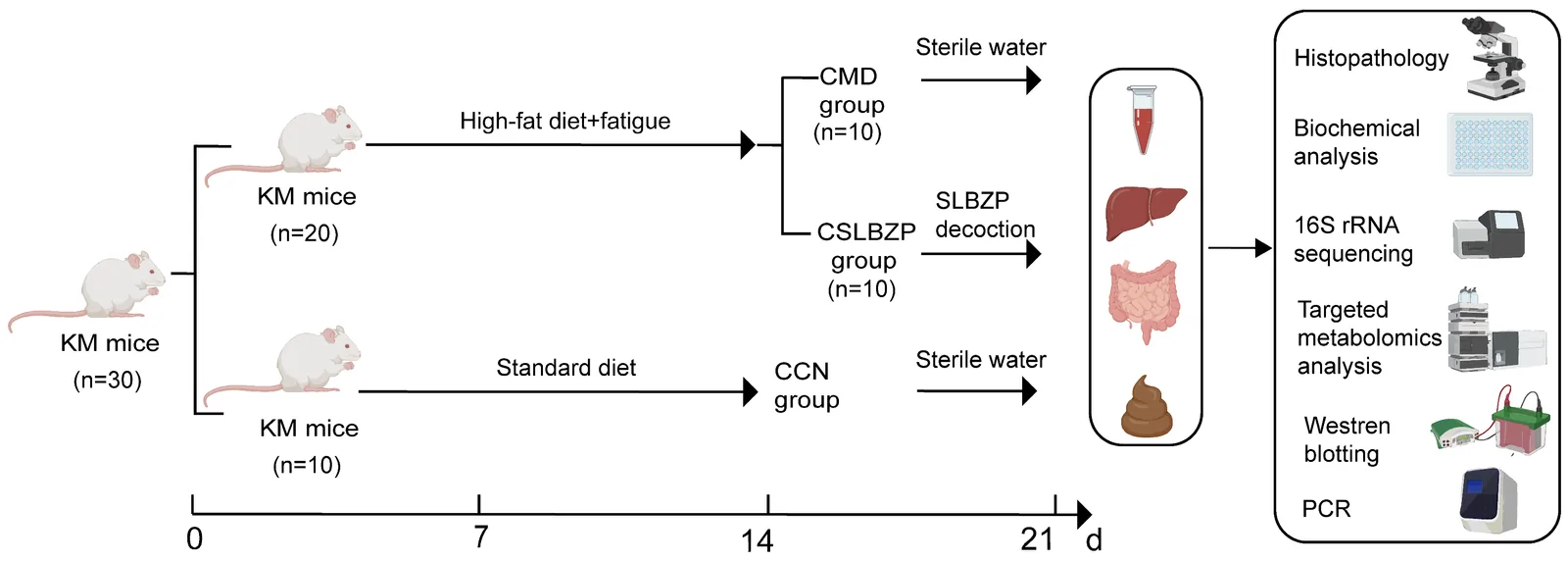
The overall experimental design and treatment schedule.

After model induction, mice in the model-induction cohort were randomly assigned to the model group (CMD, *n* = 10) and the SLBZP-treated group (CSLBZP, *n* = 10). During the 7-day treatment period, mice in the CSLBZP group received SLBZP by oral gavage at a dose of 14.56 g crude drug/kg b.w./day. Based on the adult clinical daily dose of 112 g crude herbs and a mouse conversion coefficient of 0.0026, the mouse-equivalent dose was calculated as follows: (112 g × 0.0026)/0.020 kg = 14.56 g crude drug/kg b.w./day. The SLBZP decoction was initially prepared as a stock solution containing 0.637 g crude drug/mL. Before administration, the working concentration was adjusted according to the body weight of each mouse to ensure the administration of the prescribed dose while maintaining a fixed gavage volume of 0.4 mL per mouse per administration. The daily dose was divided into two equal administrations. Mice in the CCN and CMD groups received an equal volume of sterile water twice daily. All treatments were continued for 7 consecutive days. The selected dose was based on our previous studies demonstrating its therapeutic efficacy in the same diarrhea model [24,25]. The overall experimental design and treatment schedule are shown in Figure 9.

### 4.4. Open-Field Test

After completion of the 7-day SLBZP treatment, five mice from each group were randomly selected for the OFT. The test was performed using the KSYY-OP-V4.0 real-time mouse open-field tracking and analysis system (Brainvision Biotechnology Co., Limited, Hong Kong, China). Each mouse was individually placed in the center of the arena, and spontaneous locomotor activity was recorded for 300 s. The total distance traveled and average speed were analyzed. Between trials, feces and urine were removed, and the arena was cleaned with 75% ethanol and allowed to air-dry completely before the next mouse was tested.

### 4.5. Body Weight, Food Intake, and Fecal Moisture Content

Body weight and food intake were recorded at regular intervals during the experiment. At the end of the treatment period, fresh fecal samples were collected and immediately weighed to determine the wet weight. The samples were then dried at 110 °C for 4 h and reweighed to determine the dry weight. The fecal moisture content was calculated using the formula: Fecal moisture content (%) = [(pre-drying wet weight − post-drying dry weight)/pre-drying wet weight] × 100.

### 4.6. Hematoxylin and Eosin (H&E) Staining

Liver and small intestinal tissues were dissected, gently rinsed with normal saline, and fixed in 4% paraformaldehyde for 24 h. Fixed tissues were subjected to dehydration through a graded ethanol series, paraffin embedding, and microtome (RM2016, Leica, Shanghai, China) sectioning. Following deparaffinization and rehydration, the tissue sections were stained with hematoxylin and eosin (H&E). The stained sections underwent sequential dehydration, xylene clearing, and coverslip mounting, followed by examination under an upright light microscope (Eclipse Ci, Nikon, Tokyo, Japan) to evaluate histopathological changes in the liver and small intestine.

### 4.7. Biochemical Analysis

Serum ALT and AST activities were measured using commercial assay kits (ALT: catalog No. R01502, lot No. 20250326; AST: catalog No. R01702, lot No. 20241206; Shenzhen Rayto Life and Analytical Sciences Co., Ltd., Shenzhen, China) with an automated biochemical analyzer (Chemray 240, Shenzhen Rayto Life and Analytical Sciences Co., Ltd., Shenzhen, China), according to the manufacturer’s instructions. Serum DAO concentrations were determined using a mouse-specific enzyme-linked immunosorbent assay (ELISA) kit (catalog No. JM-02511M2, lot No. 202512; Jiangsu Jingmei Biotechnology Co., Ltd., Yancheng, China) according to the manufacturer’s protocol.

### 4.8. 16S rRNA Sequencing and Analysis

Small intestinal contents were collected under aseptic conditions and subjected to 16S rRNA gene sequencing with reference to a previous study, with modifications [53]. Total microbial DNA was extracted using the MagBeads FastDNA Kit for Soil (MP Biomedicals, Irvine, CA, USA). DNA integrity was evaluated by agarose gel electrophoresis, and DNA concentration and purity were determined using a NanoDrop spectrophotometer (Thermo Fisher Scientific, Waltham, MA, USA). The V3–V4 hypervariable region of the bacterial 16S rRNA gene was amplified using the primers 338F (5′-ACTCCTACGGGAGGCAGCA-3′) and 806R (5′-GGACTACHVGGGTWTCTAAT-3′) with NEB Q5 High-Fidelity DNA Polymerase. Polymerase chain reaction (PCR) amplification was performed under the following conditions: initial denaturation at 98 °C for 5 min; 25 cycles of denaturation at 98 °C for 30 s, annealing at 52 °C for 30 s, and extension at 72 °C for 45 s; followed by a final extension at 72 °C for 5 min. The PCR products were examined by agarose gel electrophoresis, purified, and quantified using the Quant-iT PicoGreen double-stranded DNA (dsDNA) Assay Kit (Invitrogen, Carlsbad, CA, USA). Sequencing libraries were constructed using the Illumina TruSeq Nano DNA Low Throughput Library Prep Kit. Paired-end sequencing was performed on an Illumina NovaSeq platform using a 2 × 250-base pair strategy by Shanghai Personal Biotechnology Co., Ltd. (Shanghai, China). Five biological samples from each group were subjected to sequencing. Raw paired-end reads were processed using Quantitative Insights Into Microbial Ecology 2 (QIIME 2, version 2024.5). Primer sequences were removed using Cutadapt, and sequence denoising, paired-end read merging, and chimera removal were performed using the DADA2 plugin to generate amplicon sequence variants (ASVs). Taxonomic annotation was performed against the Greengenes2 database. Alpha diversity was evaluated using the Chao1, Shannon, Simpson, and observed-species indices. Differences in microbial community structure among groups were visualized using PCoA and NMDS. Microbial taxonomic composition was analyzed at the phylum and genus levels, and the relative abundances of selected bacterial taxa were compared among groups. Differentially abundant taxa were identified using LEfSe. Taxa with a linear discriminant analysis score greater than 2.0 and *p* < 0.05 were considered discriminative. The predicted functional potential of the microbial communities was assessed using Phylogenetic Investigation of Communities by Reconstruction of Unobserved States 2 (PICRUSt2). Predicted functional profiles were annotated using the Kyoto Encyclopedia of Genes and Genomes (KEGG) database and evaluated at KEGG levels 1 and 2, as well as at the individual pathway and KEGG Orthology (KO) levels.

### 4.9. Targeted Metabolomics Analysis

Colonic content samples were diluted with water and thoroughly vortexed. An aliquot of 20 μL of each diluted sample was mixed with 60 μL of acetonitrile–methanol (8:2, *v*/*v*) containing stable isotope-labeled internal standards. The mixture was sonicated for 10 min, incubated at −20 °C for 60 min, and centrifuged at 12,000 rpm for 10 min. The resulting supernatant was collected for analysis. Targeted bile acid quantification was performed by Novogene Co., Ltd. (Beijing, China) using an ExionLC™ AD ultra-high-performance liquid chromatography system coupled to a QTRAP^®^ 6500+ mass spectrometer (AB Sciex LLC, Framingham, MA, USA). Chromatographic separation was conducted on a Waters ACQUITY UPLC BEH C18 column (2.1 × 100 mm, 1.7 μm) maintained at 50 °C. Mobile phase A consisted of water containing 0.1% formic acid and 5 mM ammonium acetate, whereas mobile phase B consisted of acetonitrile. The flow rate was 0.35 mL/min, and the injection volume was 2 μL. The gradient program was as follows: 0–0.5 min, 5% B; 0.5–1.5 min, 5–30% B; 1.5–4 min, 30–37% B; 4–5 min, 37–38% B; 5–5.5 min, 38–39% B; 5.5–6 min, 39–42% B; 6–6.5 min, 42–43% B; 6.5–9.5 min, 43–50% B; 9.5–11 min, 50–60% B; 11–12 min, 60–95% B; 12–13.1 min, 95–5% B; and 13.1–15 min, 5% B. The mass spectrometer was operated in negative-ion multiple reaction monitoring (MRM) mode. The ion spray voltage was −4500 V, the curtain gas pressure was 30 psi, the source temperature was 550 °C, and ion source gases 1 and 2 were both set to 60 psi. Bile acids were quantified using authentic standards and stable isotope-labeled internal standards. Calibration curves with coefficients of determination greater than 0.99 were accepted, and the lower limit of quantification was defined at a signal-to-noise ratio of 10. Bile acid concentrations were normalized to the weight of the colonic contents and expressed as ng/g.

### 4.10. Western Blotting

Liver and ileal tissues were lysed in radioimmunoprecipitation assay (RIPA) buffer (BF0003, Wuhan Boerfu Biotechnology Co., Ltd., Wuhan, China) supplemented with protease and phosphatase inhibitors. The tissues were homogenized at 60 Hz for 120 s, and the lysates were centrifuged at 12,000 rpm for 10 min at 4 °C. Protein concentrations were determined using a bicinchoninic acid (BCA) protein assay kit (BF0026, Wuhan Boerfu Biotechnology Co., Ltd., Wuhan, China). Equal amounts of protein (25 μg per lane) were separated by 12% sodium dodecyl sulfate–polyacrylamide gel electrophoresis (SDS-PAGE) and transferred onto polyvinylidene difluoride (PVDF) membranes (W8040, BaiDaiBio, Changzhou, China) at 300 mA for 1.5 h. The membranes were blocked with 5% skim milk for 30 min at room temperature and incubated overnight at 4 °C with primary antibodies against FXR (HA721499, HuaAn Biotechnology, Hangzhou, China; 1:1000) and β-actin (AC026, ABclonal Co., Ltd., Wuhan, China; 1:50,000). After washing, the membranes were incubated with horseradish peroxidase-conjugated goat anti-rabbit immunoglobulin G (111-035-003, Jackson ImmunoResearch Laboratories, Inc., West Grove, PA, USA; 1:5000) for 30 min at room temperature. Protein bands were visualized using enhanced chemiluminescence and quantified using Image-Pro Plus software (version 6.0, Media Cybernetics, Rockville, MD, USA). Relative FXR expression in the liver and ileum was normalized to β-actin.

### 4.11. Reverse Transcription Quantitative PCR Analysis

Approximately 30–50 mg of liver or ileal tissue was homogenized in 1200 μL of TriQuick Total RNA Extraction Reagent (R1100, Solarbio, Beijing, China) using grinding beads. After incubation at room temperature for 5 min, the homogenates were centrifuged at 12,000× *g* for 10 min at 4 °C, and the supernatants were collected. Chloroform was added at 0.2 volumes, followed by vigorous vortexing, phase separation, and centrifugation under the same conditions. The aqueous phase was collected, and total RNA was precipitated with isopropanol, washed with 75% ethanol, air-dried, and dissolved in diethyl pyrocarbonate-treated water. Complementary DNA (cDNA) was synthesized using the Evo M-MLV RT Mix Kit with gDNA Clean for qPCR Ver. 2 (AG11728, Accurate Biology, Changsha, China) at 37 °C for 15 min, followed by 85 °C for 5 s. Reverse transcription quantitative polymerase chain reaction (RT-qPCR) was performed using SYBR Green Pro Taq HS Premix III (Low Rox Plus; AG11739, Accurate Biology, China). Each 20 μL reaction contained 10 μL of premix, 2 μL of primer mix, 2 μL of cDNA template, and 6 μL of nuclease-free water. The amplification conditions were as follows: initial denaturation at 95 °C for 30 s, followed by 40 cycles of 95 °C for 5 s and 60 °C for 30 s. Melting-curve analysis was performed from 60 °C to 95 °C to verify amplification specificity. The hepatic mRNA expression levels of CYP7A1, small heterodimer partner SHP, and FGFR4, together with the ileal expression of FGF15, were normalized to β-actin. Relative mRNA expression was calculated using the 2^^^−ΔΔCt method. Primer sequences are listed in Table 2.

### 4.12. Correlation Analysis

Spearman’s rank correlation analysis was performed to evaluate associations among selected bacterial taxa, FXR protein expression, and colonic bile acid concentrations. Multiple comparisons were corrected using the Benjamini–Hochberg false discovery rate (FDR) method, with *p* < 0.05 considered statistically significant.

### 4.13. Statistical Analysis

Statistical analyses were performed using SPSS 25.0 and GraphPad Prism 10.1.2. The normality of data distribution and homogeneity of variance were assessed before group comparisons. For data satisfying these assumptions, one-way analysis of variance (ANOVA) was applied; otherwise, the Kruskal–Wallis test was used. Differences were considered statistically significant at *p* < 0.05.

## 5. Conclusions

In summary, SLBZP alleviated diarrhea induced by fatigue combined with a high-fat diet in mice. SLBZP modulated the small-intestinal microbiota, with a significant increase in the relative abundance of Faecalibaculum. It also reduced total colonic bile acid levels, increased the secondary-to-primary bile acid ratio, and altered the levels of multiple bile acid species. These changes were accompanied by alterations in ileal and hepatic FXR-related signaling involved in bile acid homeostasis. Overall, the antidiarrheal effect of SLBZP may be associated with the modulation of gut microbiota, bile acid metabolism, and FXR-related signaling. These findings provide experimental evidence for further understanding the pharmacological effects of SLBZP in diarrhea.

## Figures and Tables

**Table 1:** Composition of SLBZP.

Chinese Name/Latin Name	English Name	Scientific Name	Amount (g)	Plant Part	Lot Number
Renshen/*Ginseng Radix et Rhizoma*	Ginseng	*Panax ginseng* C. A. Mey.	15	Root and rhizome	CK2531001
Baizhu/*Atractylodis Macrocephalae Rhizoma*	Largehead Atractylodes Rhizome	*Atractylodes macrocephala* Koidz.	15	Rhizome	GD25052701
Fuling/*Poria*	Poria	*Poria cocos* (Schw.) Wolf	15	Sclerotium	ZR25052602
Shanyao/*Dioscoreae Rhizoma*	Chinese Yam	*Dioscorea opposita* Thunb.	15	Rhizome	NG25042701
Lianzi/*Nelumbinis Semen*	Lotus Seed	*Nelumbo nucifera* Gaertn.	9	Seed	2504123
Baibiandou/*Lablab Semen Album*	White Hyacinth Bean	*Dolichos lablab* L.	12	Seed	QC25041801
Yiyiren/*Coicis Semen*	Coix Seed	*Coix lacryma-jobi* L. var. *ma-yuen* (Rom. Caill.) Stapf	9	Seed	RS25051402
Sharen/*Amomi Fructus*	Amomum Fruit	*Amomum villosum* Lour.	6	Fruit	QC250603
Jiegeng/*Platycodonis Radix*	Platycodon Root	*Platycodon grandiflorus* (Jacq.) A. DC.	6	Root	NG25040704
Gancao/*Glycyrrhizae Radix et Rhizoma*	Chinese Licorice	*Glycyrrhiza uralensis* Fisch.	10	Root and rhizome	TJ25051301

**Table 2:** Primer sequences used for PCR analysis.

Gene	Forward Primer (5′–3′)	Reverse Primer (5′–3′)	Product Size (bp)
β-actin	CATTGCTGACAGGATGCAGAAGG	TGCTGGAAGGTGGACAGTGAGG	138
CYP7A1	GGGCAGGCTTGGGAATTTTG	AACGCTCAGCAGTCGTTACA	116
SHP	CCAAGGAGTATGCGTACCTGAAG	GCTCCAAGACTTCACACAGTGC	126
FGFR4	TCCGACAAGGATTTGGCAGACC	TGGCGGCACATTCCACAATCAC	136
FGF15	TACGGCTGGGGCAAGATTAC	CGGATTCGGAGGAAGCAGTT	80

## Data Availability

The original contributions presented in this study are included in the article. Further inquiries can be directed to the corresponding authors.

## Supplementary Materials

The following supporting information can be downloaded at https://www.mdpi.com/article/10.3390/ph19091362/s1. Figure S1: Total ion chromatograms of SLBZP acquired in positive- and negative-ion modes; Table S1: Identification of Components in SLBZP.

## Abbreviations

The following abbreviations are used in this manuscript:

ALT	Alanine aminotransferase
ASV	Amplicon sequence variant
AST	Aspartate aminotransferase
BA	Bile acid
CCN	Normal control group
CMD	Model group
CSLBZP	SLBZP-treated group
CYP7A1	Cholesterol 7α-hydroxylase
DAO	Diamine oxidase
ELISA	Enzyme-linked immunosorbent assay
FGF15	Fibroblast growth factor 15
FGFR4	Fibroblast growth factor receptor 4
FXR	Farnesoid X receptor
H&E	Hematoxylin and eosin
HFD	High-fat diet
HRMS	High-resolution mass spectrometry
IBS-D	Diarrhea-predominant irritable bowel syndrome
KEGG	Kyoto Encyclopedia of Genes and Genomes
KM	Kunming
KO	KEGG Orthology
LC–MS/MS	Liquid chromatography–tandem mass spectrometry
LDA	Linear discriminant analysis
LEfSe	Linear discriminant analysis effect size
MRM	Multiple reaction monitoring
MS/MS	Tandem mass spectrometry
NMDS	Non-metric multidimensional scaling
OFT	Open-field test
PCA	Principal component analysis
PCoA	Principal coordinates analysis
PICRUSt2	Phylogenetic Investigation of Communities by Reconstruction of Unobserved States 2
QIIME 2	Quantitative Insights Into Microbial Ecology 2
RT-qPCR	Reverse transcription quantitative polymerase chain reaction
SHP	Small heterodimer partner
SLBZP	Shenling Baizhu Powder
SPF	Specific pathogen-free
TIC	Total ion chromatogram
UHPLC	Ultra-high-performance liquid chromatography
